# An efficient instance segmentation approach for studying fission gas bubbles in irradiated metallic nuclear fuel

**DOI:** 10.1038/s41598-023-47914-y

**Published:** 2023-12-14

**Authors:** Shoukun Sun, Fei Xu, Lu Cai, Daniele Salvato, Fidelma Dilemma, Luca Capriotti, Min Xian, Tiankai Yao

**Affiliations:** 1 University of Idaho, Idaho Falls, ID USA; 2 Idaho National Laboratory, Idaho Falls, ID USA

**Keywords:** Materials science, Nuclear energy

## Abstract

Gaseous fission products from nuclear fission reactions tend to form fission gas bubbles of various shapes and sizes inside nuclear fuel. The behavior of fission gas bubbles dictates nuclear fuel performances, such as fission gas release, grain growth, swelling, and fuel cladding mechanical interaction. Although mechanical understanding of the overall evolution behavior of fission gas bubbles is well known, lacking the quantitative data and high-level correlation between burnup/temperature and microstructure evolution blocks the development of predictive models and reduces the possibility of accelerating the qualification for new fuel forms. Historical characterization of fission gas bubbles in irradiated nuclear fuel relied on a simple threshold method working on low-resolution optical microscopy images. Advanced characterization of fission gas bubbles using scanning electron microscopic images reveals unprecedented details and extensive morphological data, which strains the effectiveness of conventional methods. This paper proposes a hybrid framework, based on digital image processing and deep learning models, to efficiently detect and classify fission gas bubbles from scanning electron microscopic images. The developed bubble annotation tool used a multitask deep learning network that integrates U-Net and ResNet to accomplish instance-level bubble segmentation. With limited annotated data, the model achieves a recall ratio of more than 90%, a leap forward compared to the threshold method. The model has the capability to identify fission gas bubbles with and without lanthanides to better understand the movement of lanthanide fission products and fuel cladding chemical interaction. Lastly, the deep learning model is versatile and applicable to the micro-structure segmentation of similar materials.

## Introduction

Next-generation advanced nuclear reactors with improved safety and economics are the future of nuclear energy for the United States and worldwide^1^. The proposed fuel forms to be utilized in advanced reactors include U metal-based alloys, Tri-structural isotropic (TRISO) particles, Uranium dioxide fuel (UO_2_), MOX fuel, and molten salts^2–5^. Although fundamental understandings of most fuel forms are well known, developing predictive models and accelerating the qualification of new fuel forms are still challenging due to the lack of quantitative data and correlation between high-level burnup/temperature and microstructure evolution^6–9^. Especially, U metal-based alloys, as complex systems, may undergo microstructure transformation, phase redistribution, and thermal property degradation in the fuel phase, and embrittlement, hardening, and corrosion of the cladding and encapsulating materials during irradiation^9–11^. The abovementioned phenomena, including microstructure transformation, phase redistribution, and thermal property changing, are strongly interconnected and form a complex multi-factor problem. This problem, in turn, makes it difficult for conventional-empirical/physical-based fuel performance models to predict fuel behavior from estimated burnup and cladding temperature accurately^12, 13^.

U-10Zr-based metallic fuel is pursued as one of the leading candidate fuel forms for next-generation sodium-cooled fast reactors for the low fabrication cost and capability to achieve a higher burnup^14, 16^. Although thousands of U-10Zr fuel rods were irritated in test reactors, such as Experimental Breeder Reactor II (EBR-II) and Fast Flux Test Facility (FFTF) from the 1960s to the 1990s^9, 14^, a lack of fundamental understanding of the nuclear fuel microstructure properties and their evolution inside a reactor decelerate the qualification for commercial use of U-10Zr fuel. One of the critical factors affecting fuel performance is the Fuel Cladding Chemical Interaction (FCCI). The majority of FCCI in the cladding, i.e., wastage, is made up of lanthanides; therefore, it is important to understand how the lanthanides migrate and what compounds form the movement of lanthanides under a temperature gradient from the hot fuel center to the cool cladding surface. The lanthanide migration and its chemical interaction with cladding are critical aspects that may result in deterioration of the cladding mechanical properties, which could threaten fuel safety^17^. Moreover, PIE on advanced irradiated U-10Zr fuels in the Advanced Test Reactor (ATR) of Idaho National Laboratory (INL) discovered that the lanthanide particles/nodules located around the periphery of the pores^18–22^. Understanding the distribution changes of the pores in the cross-section of the advanced U-10Zr fuel will provide first-of-its-kind knowledge on the lanthanide transformation, which assists in revealing the mechanism of fuel cladding chemical interactions.

**Figure 1:**
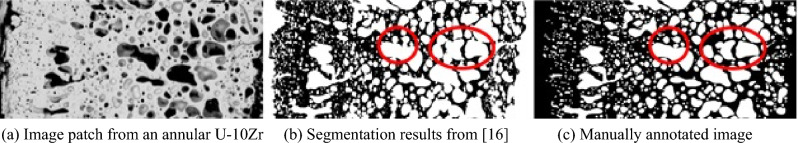
Sample results of fission gas bubble segmentation^16^.

Accurate fission gas pore detection could provide trustable morphological distribution changes of pores along thermal gradient from hot fuel region to cold cladding rim and achieve a better understanding of the lanthanide movements. Cai et al. proposed a new framework to segment and classify fission gas pores in the (U, Zr) matrix regions of a U-10Zr annular fuel^16^. The authors applied image thresholding to segment fission gas bubbles and obtained good detection performance on a dataset of ~ 800 bubbles. As shown in Fig. 1, the bubble boundaries were manually labeled Fig. 1a by a material scientist, and the final annotated images were generated by filling the bubbles' contours (white image regions in Fig. 1c). A decision tree model was trained to classify the bubbles into different categories. Although the method achieved good bubble detection performance, the method cannot separate pores well, as shown in Fig. 1b, which causes incorrect calculation of the physical properties of pores, such as size, shape, and orientation. Although the performance of machine learning (ML) models largely depends on the quality of the training data, and their interpretability may be insufficient, they typically outperform traditional approaches in terms of reliability and accuracy. Many ML approaches can be found in literature, exploring complex and large datasets to gain insights and accelerate scientific discoveries, such as accelerating testing to develop new materials^35^, automating defect detection in electron microscopy^23–25^, and so on. Existing ML-based segmentation models achieved acceptable performance on natural images^26, 27^, biomedical images^28^, and material images^29^, but detecting fission gas bubbles on fuel cross-sections is more challenging since the bubbles' appearances, including gray level, size, and shape, vary greatly. Existing image processing techniques and pre-trained ML models cannot achieve good performance. Most ML models' performance heavily depends on the amount of annotated data used at the training stage. Advanced experimental characterization tools and modern imaging routinely provide high/ultrahigh-resolution images at an ever-increasing rate and volume. However, advanced experimental characterization tools lack sufficient and high-quality annotated training data.

In this paper, a hybrid framework is proposed for more accurate and efficient fission gas bubble segmentation, and the contributions are summarized below.

1. The proposed hybrid segmentation only requires a small training set, and greatly reduces the time-consuming and expensive human efforts to manually annotate bubbles.
2. The proposed multitask instance segmentation network has a region segmentation branch and a boundary segmentation branch. It extracts and separates medium- and large-size bubbles accurately.
3. The proposed edge-based bubble segmentation approach generates accurate boundaries for small fission gas bubbles.

## Proposed Method

Three significant challenges exist when extracting bubbles from PIE images using deep learning-based approaches. First, it requires enormous time and effort to manually label, namely marking the precise boundary for each bubble, a large dataset. In our PIE images, a significant number of bubbles are unlabeled. Second, many tiny bubbles widely exist in PIE images, and annotating the regions/boundaries of these bubbles is difficult. Third, existing instance segmentation approaches could be applied to extract and separate different bubbles. However, these segmentation approaches, e.g., Mask-RCNN^30^, are inefficient and inaccurate in segmenting many closely clustered objects. To address these challenges, the bubble segmentation tasks are decomposed into two independent processing steps. In the first step, we propose a multitask instance segmentation network that is trained using a small, annotated dataset to segment medium- and large-size bubbles. The second step is unsupervised and applies an edge detection approach to extract small bubbles.

### Materials and data preparation

In recent years, INL has been the leading national laboratory for research and development (R&D) on metallic fuel. Thanks to the development of advanced characterization capabilities at the Material Fuel Complex (MFC), it is now possible to revisit the vast available PIE data accumulated in the past and the newly established PIE data ranging from sub-nanometer to micrometer to obtain new findings. In a recent study, Cai et al. proposed a segmentation framework on ~ 800 partially annotated bubbles on only three 500x-magnification image patches of a U-10Zr annular fuel named AF^16^. In this study, we collected 585 scanning electron microscope (SEM) image patches under 1000x-magnification of a partial cross-section of another advanced U-10Zr fuel named AF2^31^. The patches were collected from the hot center to the cladding. To design a DL-based model, sufficient training data with image annotations and original images were needed. Moreover, the data of the two fuels under different magnifications will reveal the features of bubbles differently, for example, size, contour, and texture, even for the same bubble as shown in Fig. 3. Under this circumstance, we developed an interactive annotation tool to label the fission gas bubbles.

### Multitask instance segmentation network (MTIS-Net) for extracting medium- and large-size bubbles

**Figure 2:**
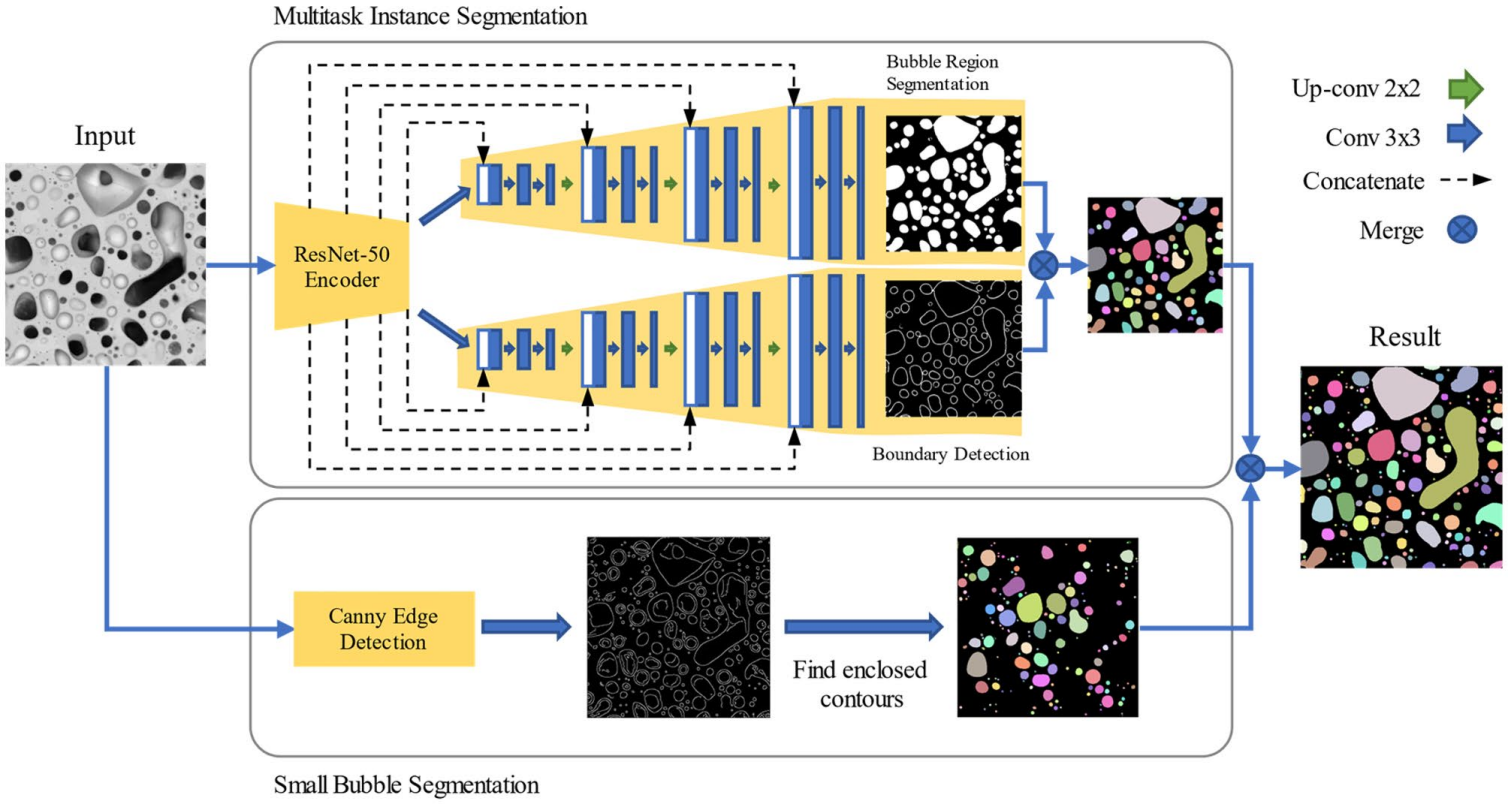
The proposed efficient instance segmentation framework.

A novel instance segmentation network is proposed to extract and separate medium- and large-size bubbles from SEM images. It treats each bubble as an instance. As shown in Fig. 2, the proposed network consists of one encoder and two decoder subnetworks. The encoder uses convolutional and pooling layers to extract meaningful features from input images at different scales. A ResNet-50 network^32^ is applied as the backbone network in the encoder. The first decoder is developed to segment bubble regions, and the second is to detect bubble boundaries. The results from the two decoders are combined to achieve an instance segmentation. The two decoders share the same feature input from the encoder. For preserving details, the intermedium feature maps of the encoder are passed to the corresponding layers in both decoders by skipping connections. These two decoders use the standard U-Net^33^ decoder architecture.

The Dice loss function^34^ is used in the bubble region segmentation branch. The Dice loss measures the quantitative difference between the region segmentation results (${\widehat{p}}_{1}$) and the ground truth (${y}_{1}$), and it is defined by

1 $${L}_{Dice}\left({y}_{1},{\widehat{p}}_{1}\right)=1-\frac{\sum 2{y}_{1}{\widehat{p}}_{1}+1}{\sum ({y}_{1}+{\widehat{p}}_{1})+1}$$

where ${y}_{1}$ is a 2D matrix that contains binary values in which value 1 denotes a bubble pixel, and value 0 represents a non-bubble pixel; and values in ${\widehat{p}}_{1}$ are the actual predictions produced by the bubble region segmentation network. The numerator and denominator are added to 1 as the smooth term to avoid division by zero.

The weighted binary cross-entropy loss function is used in the boundary detection branch, and it is given by.

2 $${L}_{WBCE}\left({y}_{2},{\widehat{p}}_{2}\right)=-\left[{w}_{0}\left(1-{y}_{2}\right)\mathrm{log}\left(1-{\widehat{p}}_{2}\right)+{w}_{1}{y}_{2}\mathrm{log}\left({\widehat{p}}_{2}\right)\right]$$

where ${y}_{2}$ is a binary map that uses 1 s to denote bubble boundary pixels; ${\widehat{p}}_{2}$ is the prediction of bubble boundaries; and ${w}_{0}$ and ${w}_{1}$ are the weights of boundary and non-boundary terms, respectively. In experiments, ${w}_{0}$ is set to 0.1, and ${w}_{1}$ is set to 0.9.

The segmentation results are produced by subtracting bubble boundaries from bubble regions. The boundaries can disconnect touching bubble regions. During the post-processing, the final bubble instances are generated by connecting bubble pixels using the 8-adjacency system^35^; and the morphological dilation operation is applied to compensate for the shrinking of bubble areas.

### Small bubble segmentation using edge detection

**Figure 3:**
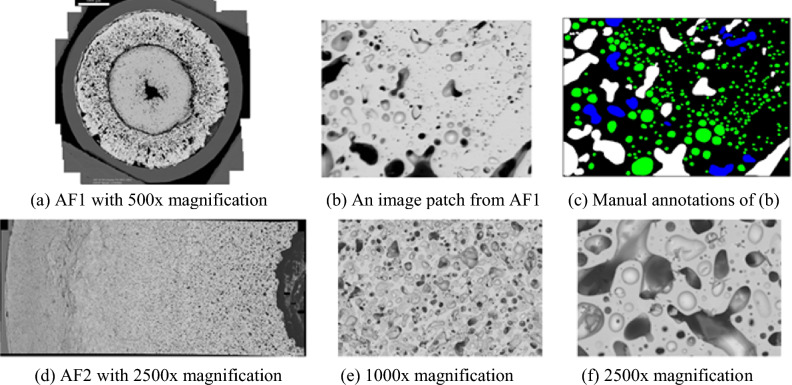
Image samples from two annular fuels under different magnifications.

**Figure 4:**
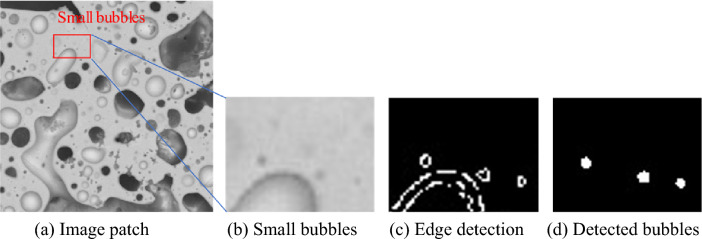
Sample results of small bubble segmentation.

As shown in Figs. 3f and 4b, small bubbles are usually presented as black or grey dots in SEM images. These dots have homogeneous interior intensities. The grey bubbles have similar intensities to the background areas; therefore, it is difficult to differentiate grey bubbles from the background by using intensity thresholds. However, the grey bubbles have dark boundaries that separate bubble regions and backgrounds, and it is more appropriate to use edge detection approaches to detect small bubbles.

In this work, we use the Canny edge detection^36^ approach which includes five steps, (1) applying a $5\times 5$ Gaussian low-pass filter ($\sigma =1$) to remove the image noise; (2) calculating the intensity gradient of the image to identify pixels with sharp intensity changes (potential edge points); (3) applying non-maximum suppression to eliminate noise; (4) applying double thresholding to determine potential edges; and (5) suppressing weak edges. The Canny edge detection approach can capture high-quality edges and mitigate the impact of image noises.

The Canny edge detection is applied to extract small bubbles. Most medium- and large-size bubbles have long, irregular-shaped, and fuzzy boundaries; and edge detection approaches can only produce small, disconnected boundary pieces, but closed boundaries could be generated for small bubbles because they have more homogeneous boundary pixels. The final bubble regions are generated by applying the flood fill algorithm^37^ to fill the closed edges. Non-closed edges are removed from the final results. Figure 4 shows sample results of small bubble segmentation.

## Experimental Results

### Setup

#### Dataset

The images are cropped into 515 × 512 non-overlapped patches. The training set contains 18 image patches and 827 annotated bubbles. It only has precise contours labeled by experts, noted as ground truths, for medium- and large-size bubbles. Due to the significant resources required, the number of medium- and large-size bubbles in the training images is small. The test set contains 24 image patches and 685 bubbles.

#### Training

We adopted a ResNet-50 backbone as the encoder because it has been demonstrated strong performance on a variety of tasks in previous studies and strikes a balance between computational efficiency and accuracy. The used backbone was pretrained using ImageNet. In training, we use an Adam^38^ optimizer with a 0.001 learning rate and train the network for 100 epochs. An exponential learning rate scheduler with $\gamma =0.97$ is used to decay the learning rate after every epoch. Training images are randomly augmented in every epoch with Gaussian blur, Gauss noise, brightness, horizontally/vertically flipping, contrast, scaling, and rotating. The batch size is set to 8.

#### Evaluation metrics

Intersection over union (IoU) is a popular metric for measuring overlap between multiple objects, especially for segmentation tasks. The higher value of IoU indicates the prediction/segmentation results align well with the actual results. In this study, both pixel-level and instance-level performances were investigated. The pixel-level evaluation assigned each pixel as pore or non-pore. Instance-level differentiates between individual pores with the pixel-level results. As the test image is partially labeled, both the instance-level recall ratio ${\mathrm{R}}_{\mathrm{iou}}^{\mathrm{I}}$ and the pixel-level ratio ${\mathrm{R}}_{\mathrm{iou}}^{\mathrm{P}}$ are used to evaluate the performance. The recall ratio is defined by Eq. (3).

3 $${\mathrm{R}}_{\mathrm{iou}}=\frac{\left|\mathrm{TP}\right|}{\left|\mathrm{P}\right|}$$

where $\left|\mathrm{TP}\right|$ denotes the number of accurately segmented bubbles (${\mathrm{R}}_{\mathrm{iou}}^{\mathrm{I}}$) or pixels (${\mathrm{R}}_{\mathrm{iou}}^{\mathrm{P}}$) and $\left|\mathrm{P}\right|$ represents the number of total bubbles or pixels in ground truths. For the instance-level recall ratio ${\mathrm{R}}_{\mathrm{iou}}^{\mathrm{I}}$, the total number of all labeled bubbles is treated as the $\left|\mathrm{P}\right|$. Each ground truth bubble is paired with a predicted bubble with the largest IoU among all predictions. The $\left|\mathrm{TP}\right|$ counts all paired predictions that have IoU values with a ground truth greater than a threshold. A set of values ($[0.5, 0.6, 0.7, 0.8, 0.9]$) are used as thresholds in experiments. The pixel-level recall, ${\mathrm{R}}_{\mathrm{iou}}^{\mathrm{P}}$, simply calculates the number of true bubble pixels over all labeled bubble pixels.

### MTIS-Net

**Figure 5:**
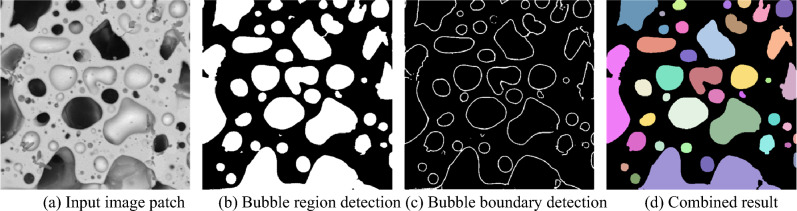
Sample results of the MTIS-Net.

Figure 5 shows an example of the outputs of the proposed MTIS-Net. The bubble region segmentation branch generates binary segmentation results for medium- and large-size bubbles. As shown in Fig. 5b, most bubbles are well-segmented, but some bubbles are connected. Figure 5c shows the segmentation results of bubble boundaries generated by the boundary segmentation branch of MTIS-Net. The final segmentation results are shown in Fig. 5d, and different bubbles are illustrated using different colors.

### Small bubble segmentation

**Figure 6:**
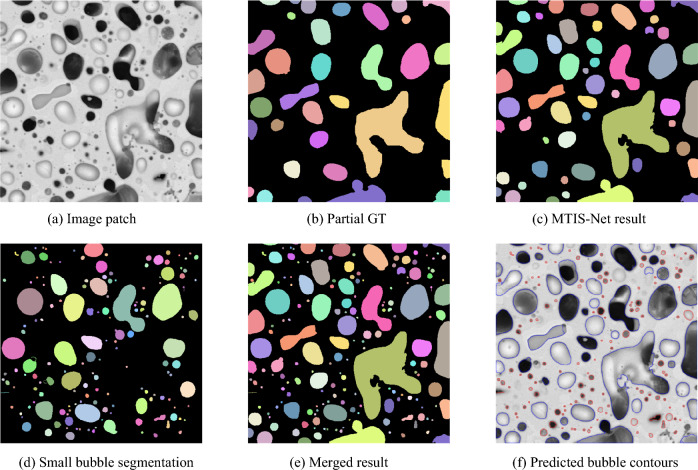
Merged Results. (**a**) Original image; (**b**) partial annotated ground truth (GT); (**c**) the result of MTIS-Net; (**d**) the result of small bubble segmentation; (**e**) the result by merging (**c**) and (**d**); (**f**) The blue contours are predicted by multi-task U-Net, and red are from small detection.

Small bubbles are segmented using the Canny edge detection approach. As shown in Fig. 6, Fig. 6a is an original image patch from the studied fuel; Fig. 6b is the manually partial annotated ground truth (GT) image of Fig. 6a,c illustrates the bubble segmentation result from the multitask model discussed in Sect. "mtis-net"; Fig. 6d shows the results of the proposed small bubble segmentation approach; and illustrates that most small bubbles have regular shapes. The merged results of the MTIS-Net and edge-based approach are shown in Fig. 6e. Figure 6f uses blue contours to show medium- and large-size bubbles identified by MTIS-Net, and red contours to demonstrate small bubbles detected by the edge-based approach.

### Overall performance

Table 1 demonstrates the instance-level recall ratios under different IoU thresholds (0.5, 0.6, 0.7, 0.8, and 0.9). In the "Bubble Numbers" table section, columns named " ≥ IoU threshold" show the number of bubbles that are successfully segmented based on specific IoU thresholds; and the "GT" column shows each image's total number of labeled objects. The "Instance-level Recall" section shows the corresponding recall values using different thresholds. In classic object localization and instant segmentation tasks, an IoU threshold of 0.5 is described as a loose criterion of the correct detection^39, 40^; and the value 0.75 is considered a “strict criterion”.

**Table 1:** Instance-level evaluation. Columns of ≥ 0.5 through to ≥ 0.9 represent the count of detected bubbles that match ground truths with an IoU equal to or larger than the threshold.

Image ID	Bubble numbers						Instance-level recall				
	≥ 0.5	≥ 0.6	≥ 0.7	≥ 0.8	≥ 0.9	GT	R^I^_0.5_	R^I^_0.6_	R^I^_0.7_	R^I^_0.8_	R^I^_0.9_
1	18	18	17	15	13	19	0.95	0.95	0.89	0.79	0.68
2	21	21	21	20	13	22	0.95	0.95	0.95	0.91	0.59
3	36	36	33	32	29	38	0.95	0.95	0.87	0.84	0.76
4	14	13	13	11	8	18	0.78	0.72	0.72	0.61	0.44
5	30	30	30	27	18	35	0.86	0.86	0.86	0.77	0.51
6	33	32	31	30	22	33	1.00	0.97	0.94	0.91	0.67
7	33	32	31	29	17	36	0.92	0.89	0.86	0.81	0.47
8	42	42	41	39	30	46	0.91	0.91	0.89	0.85	0.65
9	38	38	38	36	30	42	0.90	0.90	0.90	0.86	0.71
10	14	14	14	14	8	14	1.00	1.00	1.00	1.00	0.57
11	20	20	20	19	10	23	0.87	0.87	0.87	0.83	0.43
12	24	24	24	22	14	27	0.89	0.89	0.89	0.81	0.52
13	39	39	38	38	34	41	0.95	0.95	0.93	0.93	0.83
14	44	44	44	44	37	45	0.98	0.98	0.98	0.98	0.82
15	26	26	25	23	18	29	0.90	0.90	0.86	0.79	0.62
16	29	29	27	24	18	34	0.85	0.85	0.79	0.71	0.53
17	32	31	29	28	21	34	0.94	0.91	0.85	0.82	0.62
18	28	27	25	23	17	28	1.00	0.96	0.89	0.82	0.61
19	13	13	13	11	8	15	0.87	0.87	0.87	0.73	0.53
20	22	22	21	17	12	23	0.96	0.96	0.91	0.74	0.52
21	20	20	19	16	10	21	0.95	0.95	0.90	0.76	0.48
22	11	10	8	7	4	14	0.79	0.71	0.57	0.50	0.29
23	21	19	17	11	9	23	0.91	0.83	0.74	0.48	0.39
24	23	20	20	16	11	25	0.92	0.80	0.80	0.64	0.44
Total	631	620	599	552	411	685	0.92	0.91	0.87	0.81	0.60

Our method presents a recall ratio of 0.92 with IoU ≥ 0.5, and a recall ratio of 0.6 with IoU ≥ 0.9. With the increasing IoU thresholds, the instance level recall ratios drop slowly, which shows the precision and stability of the proposed method.

**Figure 7:**
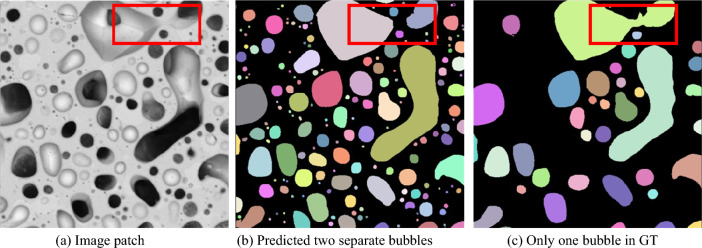
Difficulties in separating bubbles.

Even though GTs of bubble boundaries were created by experts, in specific situations, it is still challenging to appropriately define if a bubble should be separated into two. As shown in Fig. 7, the bubble inside the red box can be considered as one large bubble or two smaller bubbles. The expert labeled it a single bubble, but the model considered it two separate bubbles. Such a problem creates uncertainty in the instance-level evaluation. Hence, we also report the pixel-level recall ratio to provide a more comprehensive evaluation.

Table 2 shows the pixel-level recall ratios for each image and the entire test set. The “True Positive” column is the number of bubble pixels that are correctly classified. The "Total Positive" column represents each image’s labeled bubble pixels. The average recall ratio is 0.93. The highest and lowest recall ratio is 0.96 and 0.84, respectively.

**Table 2:** Pixel-level recall ratio.

Image ID	True positive	Total positive	Pixel-level recall
1	88,344	91,895	0.96
2	65,683	68,930	0.95
3	81,535	86,300	0.94
4	83,962	99,834	0.84
5	77,886	81,619	0.95
6	73,899	78,904	0.94
7	75,386	81,259	0.93
8	76,543	82,354	0.93
9	78,035	82,587	0.94
10	21,374	23,124	0.92
11	26,084	28,529	0.91
12	47,586	52,288	0.91
13	72,939	76,139	0.96
14	80,045	83,605	0.96
15	55,762	59,812	0.93
16	88,650	95,122	0.93
17	88,710	93,337	0.95
18	69,115	73,014	0.95
19	41,549	44,581	0.93
20	58,103	64,246	0.90
21	62,136	68,164	0.91
22	46,198	51,897	0.89
23	65,970	74,803	0.88
24	89,642	97,004	0.92
Total	1,615,136	1,739,347	0.93

### Comparison with existing work

**Figure 8:**
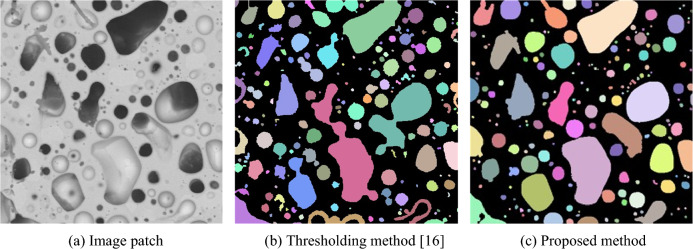
Comparison of the thresholding and proposed methods.

In the previous study by Cai et al., the bubbles were segmented with a pure image processing process that utilized the thresholding method^35^. In this section, we compare the thresholding method and the proposed method on our dataset. As shown in Fig. 8, the thresholding method tends to over-segment the bubbles, and the proposed method can generate more accurate results. Table 3 shows the instance-level recall ratios of the thresholding method and proposed method. The recall ratio of the thresholding method reaches 0.54 with the loose criterion 0.5. Meanwhile, its recall ration is only 0.03 with the 0.9 IoU threshold, and the thresholding method has poor performance in segmenting objects precisely. Compared with the instance-level recalls in Table 1, the recall ratio of the proposed method is 0.92 at IoU ≥ 0.5. The improvement of the proposed method is over 70%.

**Table 3:** Instance-level evaluation of the thresholding method ^16^ and the proposed method.

Methods	Bubble numbers						Instance-level recall				
	≥ 0.5	≥ 0.6	≥ 0.7	≥ 0.8	≥ 0.9	GT	R^I^_0.5_	R^I^_0.6_	R^I^_0.7_	R^I^_0.8_	R^I^_0.9_
Thresholding	371	283	184	98	19	685	0.54	0.41	0.27	0.14	0.03
Proposed	631	620	599	552	411	685	0.92	0.91	0.87	0.81	0.60

## Discussions

The expensive cost of preparing ground truths is one of the major challenges in ML-based fission gas bubble segmentation, especially labeling tiny bubbles. The training images are not fully labeled. Part of medium-sized, large-sized, and all small bubble areas are marked as background during training. This circumstance hinders the model from fully learning the concept of the targeted object.

Due to the incomplete ground truth, the model's performance is evaluated by recall ratio. We cannot conduct a comprehensive evaluation using more conventional instances or semantic segmentation metrics, such as mean average precision (mAP) and IoU. The drawback of recall is that it only counts on provided ground truths but cannot fully reveal the performance with the occurrence of over-segmentation.

The completely labeled ground truth with any sized bubbles is a solution to overcome the existing defects of training and evaluation. However, creating a large number of precise labels for training and evaluation is challenging. A more feasible way is to develop a semi-supervised model that can learn from incompletely labeled images. Therefore, we could train a model on a partially labeled set and evaluate it on a smaller, fully labeled set.

## Conclusion

In this study, we propose an instance-level PIE bubble segmentation approach. The proposed approach consists of a novel multitask instance segmentation network (MTIS-Net) and an image processing step for dealing with bubbles of different sizes. The proposed method obtains excellent performance with a small training set. Our model shows outstanding improvement by comparing the previously proposed thresholding method. The better performance provides more accurate quantitative results of fission gas bubbles, e.g., the distributions of different fission gas bubble classes, especially those with lanthanides. The model will be unitized on the other U-10Zr annular fuels and will contribute to building the relationship between thermal gradients and lanthanide movements. Moreover, the proposed method is promising to be applied to segmentation tasks of many materials.

## Data Availability

The datasets generated and/or analyzed during the current study are not publicly available due to the laboratory policy but should be available 3–5 years after the article is released. Currently, partial data is available from the corresponding author on reasonable request.
